# Magnetic resonance imaging of the brain in adults with severe falciparum malaria

**DOI:** 10.1186/1475-2875-13-177

**Published:** 2014-05-09

**Authors:** Richard James Maude, Frederik Barkhof, Mahtab Uddin Hassan, Aniruddha Ghose, Amir Hossain, M Abul Faiz, Ehsan Choudhury, Rehnuma Rashid, Abdullah Abu Sayeed, Prakaykaew Charunwatthana, Katherine Plewes, Hugh Kingston, Rapeephan Rattanawongnara Maude, Kamolrat Silamut, Nicholas Philip John Day, Nicholas John White, Arjen Mattheus Dondorp

**Affiliations:** 1 Centre for Tropical Medicine, Nuffield Department of Medicine, University of Oxford, Old Road, Oxford OX3 7LJ, UK; 2 Mahidol-Oxford Tropical Medicine Research Unit, Faculty of Tropical Medicine, Mahidol University, 420/6 Rajvithi Road, Rajthevee, Bangkok 10400, Thailand; 3 College of Medicine and Veterinary Medicine, University of Edinburgh, Edinburgh, UK; 4 Department of Radiology and Nuclear Medicine, VU University Medical Centre, Amsterdam, The Netherlands; 5 Chittagong Medical College Hospital, Chittagong, Bangladesh; 6 Dev Care Foundation, Dhaka, Bangladesh; 7 Center for Specialized Care and Research, Chittagong, Bangladesh; 8 Chevron Laboratory, Chittagong, Bangladesh; 9 Global Health Division, Menzies School of Health Research and Charles Darwin University, Darwin, NT, Australia

**Keywords:** MRI, Falciparum, Cerebral, Retinopathy, Pathophysiology

## Abstract

**Background:**

Magnetic resonance imaging (MRI) allows detailed study of structural and functional changes in the brain in patients with cerebral malaria.

**Methods:**

In a prospective observational study in adult Bangladeshi patients with severe falciparum malaria, MRI findings in the brain were correlated with clinical and laboratory parameters, retinal photography and optic nerve sheath diameter (ONSD) ultrasound (a marker of intracranial pressure).

**Results:**

Of 43 enrolled patients, 31 (72%) had coma and 12 (28%) died. MRI abnormalities were present in 79% overall with mostly mild changes in a wide range of anatomical sites. There were no differences in MRI findings between patients with cerebral and non-cerebral or fatal and non-fatal disease. Subtle diffuse cerebral swelling was common (n = 22/43), but mostly without vasogenic oedema or raised intracranial pressure (ONSD). Also seen were focal extracellular oedema (n = 11/43), cytotoxic oedema (n = 8/23) and mildly raised brain lactate on magnetic resonance spectroscopy (n = 5/14). Abnormalities were much less prominent than previously described in Malawian children. Retinal whitening was present in 36/43 (84%) patients and was more common and severe in patients with coma.

**Conclusion:**

Cerebral swelling is mild and not specific to coma or death in adult severe falciparum malaria. This differs markedly from African children. Retinal whitening, reflecting heterogeneous obstruction of the central nervous system microcirculation by sequestered parasites resulting in small patches of ischemia, is associated with coma and this process is likely important in the pathogenesis.

## Background

Severe falciparum malaria is a multi-organ disease with a treated mortality of 10 to 30%. There are differences in clinical presentation [1] and pathological findings [2] between adults and children. Coma (defining cerebral malaria) is one of the commonest features and is an independent risk factor for mortality in all age groups [1].

The pathogenesis of coma in malaria is not well understood. This has hampered efforts to develop adjunctive therapies to reduce mortality. Much of the current knowledge comes from autopsy studies, which only provide information on fatal cases [2]. Due to inaccessibility of the brain, studies in living subjects with cerebral malaria have been mostly limited to measurement of indirect markers [3]. Several processes are thought to be important including microvascular obstruction by sequestered erythrocytes, inflammation, endothelial dysfunction with increased blood brain barrier (BBB) permeability, and cerebral oedema. The relative contribution of these processes is unclear.

Brain imaging by computed tomography (CT) and magnetic resonance imaging (MRI) allow direct observation of the brain in living subjects. As malaria occurs predominantly in resource-limited settings, imaging studies have been limited to mostly single cases and small case series. Larger studies and more advanced MRI techniques might allow for more in-depth study of not only structural but also functional changes in the brain, including assessment of oedema, haemorrhage, ischaemia, and brain metabolism. Systematic study of cerebral malaria using advanced MRI could provide fundamental additional insights into the understanding of this disease [3].

A recent study using MRI compared findings in unconscious African children with and without malarial retinopathy [4]. In this context, absence of malarial retinopathy is thought to indicate an alternative non-malarial cause of coma. Findings of markedly increased brain volume, abnormal T2 signal intensity, and diffusion weighted imaging (DWI) abnormalities in cortical, deep gray and white matter structures were much commoner in patients with retinopathy suggesting these MRI findings are specific to malaria. In this study insights into pathogenesis were limited by lack of a control group of severe but non-comatose patients, so that the specificity of the changes could not be determined.

A prospective observational study was done using a variety of MRI techniques aiming to determine the structural and functional changes in the brain in adult patients with severe falciparum malaria.

## Methods

### Study site and patients

The study was carried out in Chittagong Medical College Hospital, Chittagong, Bangladesh, from June 2009 to August 2011. Ethical approval was obtained from the Bangladesh Medical Research Council Ethical Committee and OXTREC, the University of Oxford Tropical Research Ethics Committee.

Consecutive adult (≥16 years) patients with slide-confirmed severe falciparum malaria, according to modified WHO criteria [5], were eligible for inclusion. Cerebral malaria was defined as Glasgow Coma Score (GCS) <11 out of 15 in the absence of hypoglycaemia (<2.2 mmol/L). Severe but non-cerebral malaria was defined as GCS ≥ 11 plus one or more of the other severity criteria listed in Table 1.

**Table 1:** Presenting severity signs of enrolled patients

	**Number (%)**	
	**(n = 43)**	
Cerebral malaria (GCS < 11)	31	(72%)
Venous lactate >4 mmol/l	20	(47%)
Jaundice (bilirubin >2.5 mg/dl + parasites >100,000/mm3)	9	(21%)
Hyperparasitaemia (>10%)	8	(19%)
Generalized convulsions (≥2 in 24 hours)	7	(16%)
Acidosis (venous bicarbonate <15 mmol/l)	6	(14%)
Renal failure (creatinine >3 g/dL or anuria)	5	(12%)
Severe anaemia (Hct < 20% + parasites > 100,000/mm3)	3	(7%)
Spontaneous bleeding	1	(2%)
Hypoglycaemia (blood glucose <40 mg/dl)	1	(2%)
Pulmonary oedema	0	(0%)
Shock (systolic BP < 80 + cool peripheries)	0	(0%)

Patients were excluded if they died before imaging could be done or if MRI was deemed unsafe (due to shock (systolic blood pressure <80 mm Hg with cool extremities), hypoglycaemia (blood glucose <2.2 mmol/L), or signs of respiratory insufficiency (respiratory rate >32/min, nailbed oxygen-saturation <90% by pulse oximetry, signs of pulmonary oedema on physical exam or chest x-ray)) or the presence of metallic devices, pregnancy or lactation. Those with documented allergy to MRI contrast media or acute renal failure (serum creatinine >1.4 mg/dL and estimated glomerular filtration rate (eGFR) <30 mL/min [6]) did not receive contrast media.

### Study procedures

On admission a full history and examination were carried out. Blood samples were taken for haemoglobin, haematocrit, parasite count, platelet count, white cell count, glucose, plasma lactate, full biochemistry and *Plasmodium falciparum* histidine rich protein 2 (PfHRP2; as a marker of parasite biomass [7]). All patients underwent retinal photography (using a Kowa Genesis D retinal camera, Kowa Company Ltd., Tokyo, Japan) through dilated pupils on admission with ‘blinded’ analysis by a single observer (RJM) grading severity findings according to published classification criteria [8]. From 2011 onwards patients also underwent orbital ultrasound (Accutome B-scan Plus, Accutome Inc., Malvern, PA, USA) to determine optic nerve sheath diameter as a marker of intracranial pressure (ICP). Each ultrasound was recorded on video and later scored by two blinded observers (RJM and RRM) according to previously described methods [9].

### MRI scanning

Imaging of the brain was performed using either a 1.5T (Magnetom Avanto, Siemens AG, Erlangen, Germany) or a 0.3T (Airis II, Hitachi Medical Corporation, Tokyo, Japan) MRI scanner. The 1.5T scanner was available from 2010 onwards. Availability of gadolinium contrast medium was limited throughout the study. Scanning was done on the 1st day of admission when possible (up to a maximum 48 hours after admission).

The MRI sequences performed were as follows: for both 0.3 and 1.5T scans: 1. Sagittal T1-weighted images to identify midline and Anterior-Posterior Commissure (AC-PC) line for slice positioning and to evaluate swelling and major venous sinus patency; 2. Axial T2-weighted and Fluid Attenuated Inversion Recovery (FLAIR) turbo spin echo for lesion identification; 3. Gradient Echo (GRE) for micro-haemorrhages. For 1.5T scans: 4. Axial trace-diffusion weighted imaging (DWI) (b-values 0, 500, 1000 s/mm2); and 5. Single-voxel Magnetic Resonance Spectroscopy (MRS) of the parietal grey matter (2×2×2 cm) using Short Echo Time (TE)-Stimulated Echo Acquisition Mode (STEAM). 6. Axial T1-Spin Echo (T1-SE) after contrast (dimeglumine gadopentetate, 4690 mg in 10 ml, Bayer Schering Pharma AG, Berlin, Germany), in stable patients with normal renal function on enrollment repeated after 10 minutes. Pulse sequences included T2, FLAIR, T1 and T2* gradient echo.

Image analysis consisted of: 1. Visual rating of infarcts and white matter lesions on FLAIR/T2; 2. Determination of cytotoxic edema from DWI with confirmation by ADC maps; 3. Assessment of cerebral swelling from sagittal T1 and axial FLAIR/T2 as none, mild (just discernable), moderate (clearly evident without mass effect) or severe (extensive with mass effect); 4. Detection of haemorrhage by susceptibility effect on T2 or bright signal on T1 and venous patency; 5. Calculation of metabolite ratios (Choline/creatinine, N-acetyl aspartate (NAA)/Creatinine and Lactate/Creatinine) using MRS on a 2 cm^3^ cube of parietal cortex; and 6. Determination of blood brain barrier (BBB) leakage from T1-SE scans. Image analysis was done by a single expert (FB) blinded to all demographic and clinical information.

### Drug and supportive treatments

Antimalarial treatment was with intravenous artesunate followed by artemether-lumefantrine when the patient was recovering, with supportive treatment in accordance with 2006 and 2010 WHO guidelines [10,11] and local hospital guidelines, although access to mechanical ventilation and renal replacement therapy was limited.

### Statistical analysis

Numbers of patients were compared using Chi-square with Yate’s correction or Fisher’s exact tests as appropriate. When appropriate, data were log transformed to obtain a normal distribution. Normally distributed data were compared using Student’s *t* test. The Mann–Whitney *U* test was used for unpaired nonparametric data. The level of significance was p < 0.05.

## Results

During the study period, 97 adults were admitted with severe falciparum malaria. No scanner was available for 35 patients, and 12 died before having MRI. MRI was contraindicated in 5 with respiratory insufficiency and 2 with hypoglycaemia. The remaining 43 patients had MRI scans. In 9/43 (21%) image quality was reduced due to movement of the patient during the scan.

The 1.5T scanner was used on 26/43 (60%) patients (17 cerebral, 9 non-cerebral) and 0.3T on 17/43 (40%) patients (14 cerebral and 3 non-cerebral). The median (interquartile range (IQR)) time from enrollment to MRI scan was 22.6 (3.7-29.5) hours. 17/43 (40%) scans (13 with the 1.5T scanner) were done within the first 10 hours of admission.

Of those enrolled, 31/43 (72%) had cerebral, 12/43 (28%) non-cerebral but severe disease and 7/43 (16%) had ≥2 convulsions in the 24 hours prior to admission. No patient had any focal neurological signs. Infections were fatal in 12/43 (28%). Median (range) age was 30 (16–75) years; 35/43 (81%) were male. Severity criteria on enrollment are listed in Table 1.

### MRI

**Figure 1:**
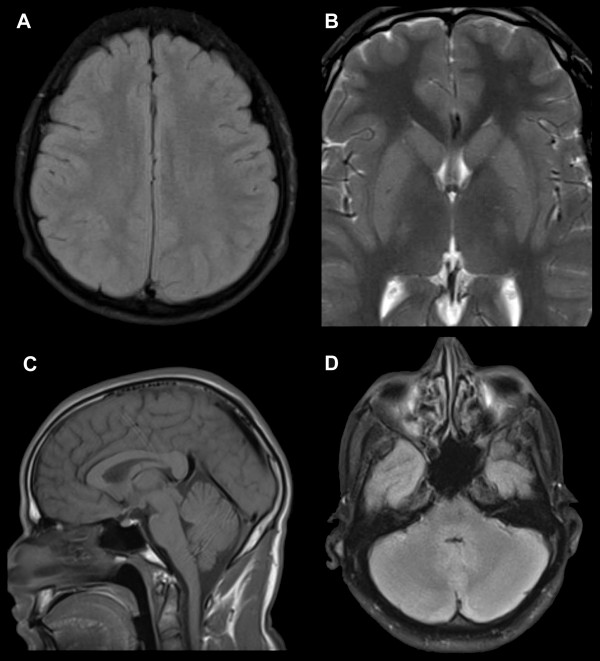
**Examples of MRIs from 4 patients with severe malaria. A** diffuse moderate supratentorial swelling on FLAIR with obliteration of sulcal pattern, **B** bilateral swollen striatum on T2 with mildly increased signal intensity and blurred borders, **C** diffuse mild supratentorial and marked poster fossa swelling on T1 and **D** marked posterior fossa swelling and mild signal increase on FLAIR.

MRI findings are summarized in Additional file 1: Table S2. Examples of MRI findings are shown in Figure 1. Overall, 34/43 (79%) patients had abnormalities on MRI; 12/17 (71%) by 0.3T and 22/26 (85%) by 1.5T scan (p = 0.44). These abnormalities were found in a variety of anatomical sites: in the supratentorial region (ST) in 22/43 (51%) (including basal ganglia (BG) in 9/43 (21%)), and posterior fossa (PF) in 16/43 (37%). There were no differences in MRI findings between individuals with cerebral and non-cerebral malaria and no differences between fatal and nonfatal infections. This was true for all MRI sequences and at all anatomical locations.

The commonest abnormalities were mild degrees of diffuse swelling in the supratentorial region and/or posterior fossa. In 19/22 (86%), this swelling was mild, 2/22 moderate (1 PF and 1 ST) and 1/22 marked (PF). Diffuse swelling was present in both ST and PF in 12/22 (55%), 5/22 only in the PF and 5/22 only in the ST including 2/5 only in the BG. There was no supra- or infratentorial brain herniation visible in any of the patients. On T2 weighted and FLAIR imaging the swollen areas showed a normal signal in 16/22 (73%) indicating the swelling is not due to extracellular (vasogenic) oedema. Of the other 6 cases, 4 had a high signal only in the basal ganglia whereas swelling of the brain was diffuse in both the ST and PF in 2 of these cases. In addition 7/13 (54%) patients had normal DWI in the swollen areas indicating that most of the swelling was not due to cytotoxic oedema. Gadolinium contrast was given to 5 patients, 4 patients with swelling and 1 without. Two of those with swelling had venous congestion, both of these had coma, one of whom died. Of those with swelling but no venous congestion one had coma and one did not. The patient without swelling or venous congestion was comatose on enrollment.

Overall, high signal on T2/FLAIR was present in 11/43 (26%) patients with most having focal lesions; 4 of these had lesions only in the basal ganglia (2 diffuse and 2 in striatum only), 2 only in the globus pallidus, 1 in globus pallidus and pons, 1 in corpus callosum, 1 widespread in cerebral cortex, 1 in parietal cortex and 1 in the cerebellum.

On DWI, 8/23 (31%) were abnormal with low ADC. 7/8 (88%) of these had coma (1/8 versus 7/8, p = 0.18) and 3/8 (38%) were fatal (3/8 versus 5/8 p = 0.66). High signal was present diffusely in the cerebral cortex in 3/23 (13%) patients (1 throughout the cortex and basal ganglia, 1 throughout the cortex and around the superior colliculus and 1 only subtle changes in the parieto-occipital cortex and the putamen). 2/3 of those with diffuse high cortical signal on DWI had coma. Another patient had subtle high signal throughout the cerebellum. Isolated focal high signal was also seen on DWI in the globus pallidus (1 patient), putamen (1 patient) and splenium (2 patients). Abnormal areas on DWI corresponded to high signal on T2/FLAIR in 5 patients (1 diffuse cerebral cortex and superior colliculus, 1 splenium of corpus callosum, 1 globus pallidus, 1 striate and 1 cerebellum).

On magnetic resonance spectroscopy (MRS), 10/14 (71%) had abnormalities: 5 mildly raised choline/creatinine ratios, 5 mildly raised lactate/creatinine ratios and none had raised N-acetylaspartic acid/creatinine ratios. Raised lactate was not associated with diffuse changes on DWI. There was no difference in mean (95%CI) peripheral blood lactate in those with (4.48 [3.26-5.70] mmol/L) and without (5.16 [3.4-6.92] mmol/L, p = 0.61) a raised lactate/creatinine ratio on MRS. None of the patients had haemorrhages or microhaemorrhages on GRE and no incidences of cerebral venous thrombosis were detected.

For the seven patients presenting with ≥2 convulsions in the 24 hours prior to admission, the MRI findings were not different to those without convulsions: 3/7 had swelling, 1 mild in ST and PF; 1 mild in ST, basal ganglia and PF with increased signal on DWI, lactate on MRS and venous congestion post contrast; and the other with moderate in ST plus increased signal on FLAIR and DWI.

### ONSD

Measurement of ONSD was done in 14 patients. Median (range) ONSD was 4.77 (4.32-4.96) mm. In those with swelling on MRI (n = 10), ONSD was 4.77 (4.38-4.96) mm *vs* 4.62 (4.32-4.94) mm in those without swelling (n = 4, P = 0.67). Using a cut-off of 4.75 mm for this population, [9] 8/14 patients had abnormally enlarged ONSD, 6/10 (60%) with swelling and 2/4 (50%) without (P = 0.61). There was no difference in ONSD between those with and without coma (p = 0.60) and those with fatal and nonfatal infections (p = 0.69).

### Retinopathy

**Figure 2:**
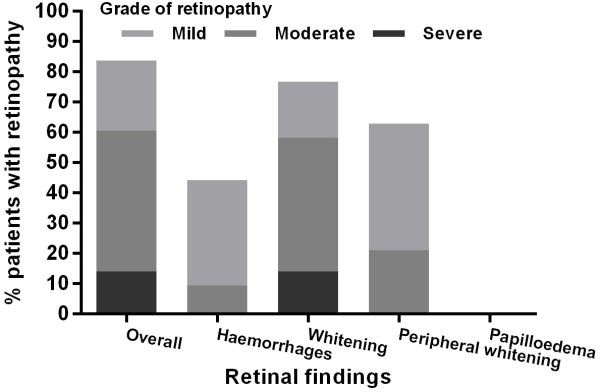
Summary of retinal findings.

**Figure 3:**
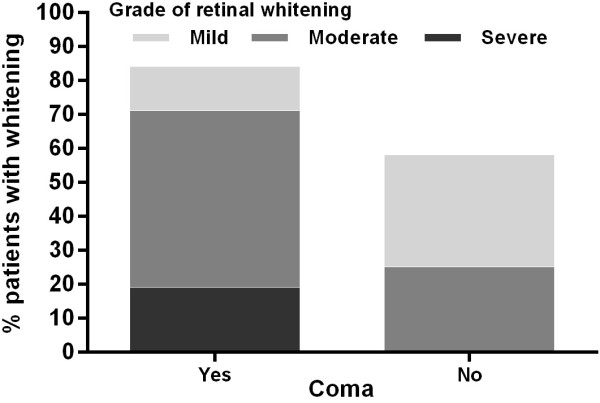
Proportion of patients with and without coma with different grades of retinal whitening.

**Figure 4:**
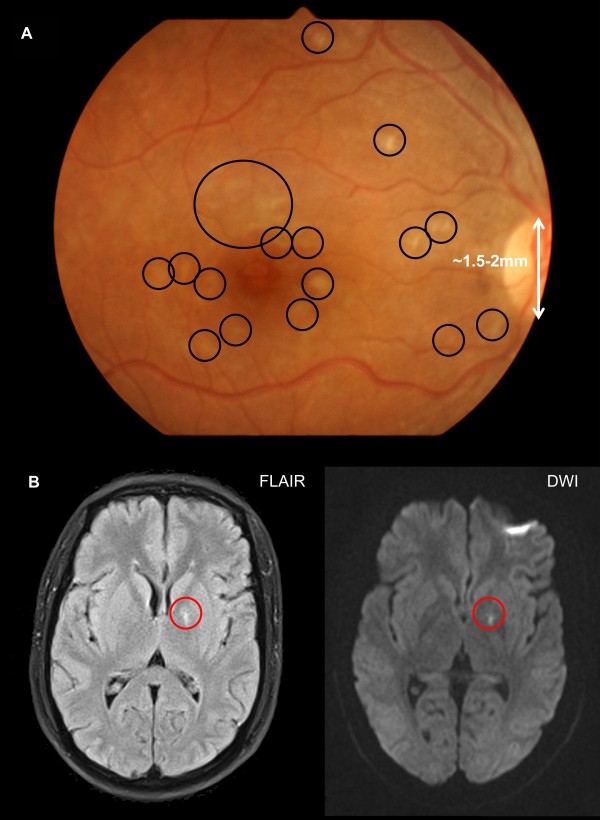
**Retinal photograph and MRI from patient with cerebral malaria (GCS = 8 on enrollment), hyperlactaemia and 98% sequestered biomass. A** the retina has typical lesions of retinal whitening (black circles) in the macula and fovea. Using the vertical optic disc diameter (white) as reference [12], each lesion of whitening is estimated at around 0.2-0.5 mm diameter. **B** on MRI the only abnormality was high signal in the globus pallidus on T2/FLAIR (left) and DWI (right).

Retinal photography was performed in all patients (Figure 2). 36/43 (84%) had malarial retinopathy; 26/43 (60%) had moderate to severe retinal changes, which were more common in those with coma (22/31 (71%)) than those without coma (3/12 (25%), p = 0.014, Figure 3). None had papilloedema. Moderate-severe peripheral retinal whitening was only present in those with coma (9/31 (29%)) and not in those without (0/12 (0%), p = 0.044). An example of this is shown in Figure 4. There were no associations between findings on MRI and presence or absence of different features of retinopathy. Moderate-severe retinal whitening was not associated with raised lactate on MRS (4/5 (20%) with raised lactate *vs* 5/10 (50%) without, p = 0.58).

### Sequestered biomass

There was no difference in median (range) plasma PfHRP2 concentration, total calculated parasite burden [7] or calculated proportion of sequestered biomass between those with swelling on MRI and those without (p = 0.27, 0.11 and 0.14). Those with venous congestion after contrast and moderate to severe cortical swelling did not have a higher sequestered biomass than the rest of the cohort.

## Discussion

This study describes a wide variety of mostly subtle changes on brain MRI in adults with severe falciparum malaria. None of the changes were more frequent in those with coma compared to severe disease without coma, or in patients with fatal disease compared to survivors. Diffuse mild brain swelling was present in the majority without evidence of diffuse cerebral oedema, either cytotoxic (on DWI) or vasogenic (T2 and FLAIR) in most. This swelling was probably at least partly due to venous congestion of the sequestered parasitized red cell mass causing increased cerebral blood volume, whereas no venous thrombosis was detected. The observed brain swelling was considered insufficient to cause coma and was in addition not specific to coma or fatal disease in this series and not correlated to ONSD. The MRI changes thus appear not to be in a causal relationship with observed neurological symptoms. The DWI abnormalities observed coincided with low ADC and most likely represented cytotoxic oedema, perhaps related to ischaemia, or possibly acute demyelination.

In this study, most patients had retinal whitening which was mostly diffuse small patches and more common and severe in those with coma. The retina is part of the central nervous system (CNS) and whitening is thought to be due to ischaemia as a result of heterogeneous obstruction of the microvasculature by sequestered parasites [13]. As the retinal vasculature is very similar to that in the brain, it strongly suggests that ischaemia would also occur in the brain. In autopsy studies the amount of sequestration and microvascular congestion in the brain has been shown to correlate with coma in malaria [14]. On brain MRI in the present study there was a lack of diffuse ischaemic changes on DWI and complete absence of haemorrhages on GRE. This may be because MRI is insufficiently sensitive to detect small lesions of very focal ischaemia. Retinal lesions seen in this study were typically 0.2-0.5 mm in diameter and brain haemorrhages on post-mortem studies of fatal malaria are typically microhaemorrhages [15]. 1.5T MRI is limited to 1 mm for T1 and T2 and 2 mm or more for DWI [16]. Ultrahigh-field MRI at 7 or 8T would be required to show lesions of this magnitude on T2/FLAIR imaging [17] and 1 mm on DWI [18]. Such scanners are generally not available in the resource poor areas where malaria is common.

A minority of patients had raised lactate on MRS, although raised CSF lactate in cerebral malaria is common [19]. MRS in this study was limited to a single voxel in the parietal cortex but ischaemic lesions were predominant in the brainstem and basal ganglia. Systemic markers of ischaemia (blood lactate) and sequestered biomass did not correlate with the findings on MRI. This may reflect the heterogeneous distribution of parasite sequestration in different organs in the body as shown in autopsy studies [20]. Explanations for this include differences in endothelial cell surface receptors in different tissues [21] and between individuals.

The MRI and retinal findings in this study contrast to those seen in previous studies in African children [4]. In both populations, lesions in the brain in severe malaria were found in a broad range of anatomical locations. However, the type and severity of abnormalities seen was markedly different. The most common abnormality in Malawi was basal ganglia lesions, present in >80% compared to 23% in the present study. Brain swelling was much less severe in the present study than in African children [4,22]. In previous imaging studies most adults with cerebral malaria had little evidence of cerebral oedema [23,24], or showed mostly moderate brain swelling not correlating with coma depth [25]. High signal on T2 associated with thickening of the supratentorial cortex was present in the majority of Malawian children [4]. In contrast to Bangladeshi adults, this suggests the swelling in Malawian children was at least partly due to oedema. In many of these children, the T2 changes were confluent and in some associated with diffuse abnormalities on DWI. These larger lesions were not seen in adults in the present study and this mirrors differences in the lesions seen in the retina; confluent patches of retinal whitening being common in African children with cerebral malaria but absent in Bangladeshi adults [26,27].

In adults, a slight increase in brain volume has been attributed to increased intracranial blood volume probably as a consequence of sequestration of parasitized erythrocytes [24]. The present study appears to confirm this by finding brain swelling and venous congestion without signs of increased ICP. Raised intracranial pressure in children [28] and the extent of brain swelling on CT in adults [25,29] are unrelated to mortality and depth of coma. Mannitol to reduce ICP in cerebral malaria did not improve outcome in adults [25] or children [30]. The exact role of raised ICP in the pathogenesis of cerebral malaria is unclear, but seems to play only a minor role in adults. Rather than a primary cause for coma it is more likely a feature developing in the later stages of the disease.

This study had several limitations. It was not possible to perform all MRI sequences in all patients due to limited availability of scanners and patients being too unwell or restless. MRI was not performed in half of the fatal cases who died shortly after admission, which could have confounded the selection of patients. However, as most MRIs were done on the day of admission, and all within 48 hours, it seems unlikely that this will have had a major effect on the findings. The scans were assessed by a single observer. Interobserver variability could thus not be quantified.

The mechanisms of coma and death in malaria are probably multifactorial and individual factors might contribute to different degrees between individuals. Subtle variations in the amount and location of sequestration and swelling may lead to coma in some individuals but not be apparent on MRI. Sequestration could target neurotoxic substances produced by the parasite. Changes in areas that do not determine consciousness could be obvious on MRI but not result in coma. In addition, metabolic disturbance outside the brain may cause coma and death with a normal MRI appearance.

MRI has great potential to further elucidate the pathogenesis of coma and death in malaria. Future studies should use MRS to study metabolic disturbance in different parts of the brain and gadolinium contrast to quantify cerebral perfusion and map venous congestion. The availability of increasingly sophisticated scanning software and more powerful scanners should greatly assist in these efforts.

## Conclusions

A variety of abnormalities were identified with different MRI techniques in adult patients with severe falciparum malaria. Mild brain swelling likely caused by venous congestion was common but much less severe than previously seen in Malawian children. MRI findings in non-comatose individuals with severe malaria have not previously been examined. None of the observed changes on MRI were specific to patients with coma or fatal disease suggesting the processes they represent are not central to their pathogenesis.

## Abbreviations

ADC: Apparent diffusion coefficient; CNS: Central nervous system; CT: Computed tomography; DWI: Diffusion weighted imaging; eGFR: Estimated glomerular filtration rate; FLAIR: Fluid attenuated inversion recovery; GRE: Gradient echo; GCS: Glasgow coma scale; ICP: Intracranial pressure; MRI: Magnetic resonance imaging; MRS: Magnetic resonance spectroscopy; ONSD: Optic nerve sheath diameter; PF: Posterior fossa; PfHRP2: *Plasmodium falciparum* histidine rich protein 2; ST: Supratentorial region; WHO: World Health Organization.

## Competing interests

The authors do not have a commercial or other association that might pose a competing interest.

## Authors’ contributions

RJM designed the study, led the field work, collected and analysed data and wrote the report. FB designed the study, interpreted the MRI images and wrote the report. AMD designed the study, wrote the report and supervised the work. MUH, AG, AH, EC, RR and AAS collected data, supervised the fieldwork and wrote the report. PC, KP, HK, RRM and KS collected data and wrote the report. MAF, NPJD and NJW wrote the report and supervised the work. All authors approved the final version of the manuscript.

## Supplementary Material

Additional file 1: Table S2 MRI findings in 43 Bangladeshi patients with severe and cerebral malaria. For 1.5T MRI, n = 26 and 0.3T n = 17. Click here for file
