# Supplementary material for: Magnetic resonance imaging of the brain in adults with severe falciparum malaria
Source: Malar J. 2014 May 9;13:177. doi: 10.1186/1475-2875-13-177 (PMC4024626; doi:10.1186/1475-2875-13-177)
Supplement: Additional file 1: Table S2 — MRI findings in 43 Bangladeshi patients with severe and cerebral malaria. For 1.5T MRI, n = 26 and 0.3T n = 17. [file 1475-2875-13-177-S1.docx]

**Table 2.** MRI findings in 43 Bangladeshi patients with severe and cerebral malaria. For 1.5T MRI, n=26 and 0.3T n=17.

| MRI abnormality | Scanner | Location/type | Overall | | Cerebral | | Non-cerebral | | P |  | Died | | Survived | | P |
| --- | --- | --- | --- | --- | --- | --- | --- | --- | --- | --- | --- | --- | --- | --- | --- |
|  | (Tesla) |  | (n=43) | | (n=31) | | (n=12) | |  |  | (n=12) | | (n=31) | |  |
| All | 1.5+0.3 | All | 34 | (79%) | 24 | (77%) | 10 | (83%) | 1 |  | 9 | (75%) | 25 | (81%) | 0.69 |
|  | 1.5 | All | 22 | (85%) | 14 | (82%) | 8 | (89%) | 1 |  | 6 | (86%) | 16 | (84%) | 1 |
|  | 0.3 | All | 12 | (71%) | 10 | (71%) | 2 | (67%) | 1 |  | 3 | (60%) | 9 | (75%) | 0.6 |
|  |  |  |  |  |  |  |  |  |  |  |  |  |  |  |  |
| High signal | 1.5+0.3 | All | 11 | (26%) | 8 | (26%) | 3 | (25%) | 1 |  | 2 | (17%) | 9 | (29%) | 0.66 |
| on T2/FLAIR | 1.5 | All | 7 | (27%) | 5 | (29%) | 2 | (22%) | 1 |  | 1 | (14%) | 6 | (32%) | 0.63 |
|  | 0.3 | All | 4 | (24%) | 3 | (21%) | 1 | (33%) | 1 |  | 1 | (20%) | 3 | (25%) | 1 |
|  |  |  |  |  |  |  |  |  |  |  |  |  |  |  |  |
| Swelling | 1.5+0.3 | All | 23 | (53%) | 17 | (55%) | 6 | (50%) | 1 |  | 7 | (58%) | 16 | (52%) | 0.74 |
|  | 1.5+0.3 | Supratentorial | 17 | (40%) | 13 | (42%) | 4 | (33%) | 0.73 |  | 5 | (42%) | 12 | (39%) | 1 |
|  |  | Posterior fossa | 18 | (42%) | 12 | (39%) | 6 | (50%) | 0.52 |  | 7 | (58%) | 11 | (35%) | 0.06 |
|  |  | ST and PF | 11 | (26%) | 7 | (23%) | 4 | (33%) | 0.47 |  | 5 | (42%) | 7 | (23%) | 0.19 |
|  |  | Basal ganglia | 2 | (5%) | 2 | (6%) | 0 | (0%) | 1 |  | 0 | (0%) | 2 | (6%) | 1 |
|  | 1.5 | All | 14 | (54%) | 10 | (59%) | 4 | (44%) | 0.63 |  | 6 | (86%) | 8 | (42%) | 0.08 |
|  | 0.3 | All | 9 | (53%) | 7 | (50%) | 2 | (67%) | 1 |  | 1 | (20%) | 8 | (67%) | 0.13 |
|  |  |  |  |  |  |  |  |  |  |  |  |  |  |  |  |
| Atrophy | 1.5+0.3 | All | 3 | (7%) | 1 | (3%) | 2 | (17%) | 0.18 |  | 0 | (0%) | 3 | (10%) | 0.55 |
|  | 1.5 | All | 3 | (12%) | 1 | (6%) | 2 | (22%) | 0.27 |  | 0 | (0%) | 3 | (16%) | 0.54 |
|  | 0.3 | All | 0 | (0%) | 0 | (0%) | 0 | (0%) | 1 |  | 0 | (0%) | 0 | (0%) | 1 |
|  |  |  |  |  |  |  |  |  |  |  |  |  |  |  |  |
| Haemorrhage | 1.5+0.3 | All | 0 | (0%) | 0 | (0%) | 0 | (0%) | 1 |  | 0 | (0%) | 0 | (0%) | 1 |
|  |  |  |  |  |  |  |  |  |  |  |  |  |  |  |  |
| High signal on DWI | 1.5 only | *Total done* | *23* |  | *15* |  | *8* |  |  |  | *7* |  | *16* |  |  |
|  |  | Abnormal | 8 | (35%) | 7 | (47%) | 1 | (13%) | 0.18 |  | 3 | (43%) | 5 | (31%) | 0.66 |
|  |  |  |  |  |  |  |  |  |  |  |  |  |  |  |  |
| MRS high ratio | 1.5 only | *Total done* | *14* |  | *9* |  | *5* |  |  |  | *7* |  | *7* |  |  |
|  |  | Choline/creatinine | 5 | (36%) | 2 | (22%) | 3 | (60%) | 0.27 |  | 3 | (43%) | 2 | (29%) | 1 |
|  |  | NAA/creatinine | 0 | (0%) | 0 | (0%) | 0 | (0%) | 1 |  | 0 | (0%) | 0 | (0%) | 1 |
|  |  | Lactate/creatinine | 5 | (36%) | 4 | (44%) | 1 | (20%) | 0.58 |  | 2 | (29%) | 3 | (43%) | 1 |
|  |  |  |  |  |  |  |  |  |  |  |  |  |  |  |  |
| Gadolinium enhanced | 1.5 only | *Total done* | *5* |  | *4* |  | *1* |  |  |  | *2* |  | *3* |  |  |
|  |  | Venous congestion | 2 | (40%) | 2 | (50%) | 0 | (0%) | 1 |  | 1 | (50%) | 1 | (33%) | 1 |
